# A simple semi-automated home-tank method and procedure to explore classical associative learning in adult zebrafish

**DOI:** 10.3758/s13428-023-02076-7

**Published:** 2023-02-22

**Authors:** Alexis Buatois, Zahra Siddiqi, Sadia Naim, Tulip Marawi, Robert Gerlai

**Affiliations:** 1 Department of Psychology, University of Toronto Mississauga, Rm CCT4004, 3359 Mississauga Road, Mississauga, Ontario L5L 1C6 Canada; 2 Institute of Neuroscience and Physiology, Department of Neurochemistry and Psychiatry, University of Gothenburg, Su Sahlgrenska, 41345 Göteborg, Sweden; 3 Department of Cell and Systems Biology, University of Toronto, 25 Harbord St, Toronto, Ontario M5S 3G5 Canada

**Keywords:** Zebrafish, Cognition, Elemental learning, Automated device, Food conditioning

## Abstract

The zebrafish is a laboratory species that gained increasing popularity the last decade in a variety of subfields of biology, including toxicology, ecology, medicine, and the neurosciences. An important phenotype often measured in these fields is behaviour. Consequently, numerous new behavioural apparati and paradigms have been developed for the zebrafish, including methods for the analysis of learning and memory in adult zebrafish. Perhaps the biggest obstacle in these methods is that zebrafish is particularly sensitive to human handling. To overcome this confound, automated learning paradigms have been developed with varying success. In this manuscript, we present a semi-automated home tank-based learning/memory test paradigm utilizing visual cues, and show that it is capable of quantifying classical associative learning performance in zebrafish. We demonstrate that in this task, zebrafish successfully acquire the association between coloured-light and food reward. The hardware and software components of the task are easy and cheap to obtain and simple to assemble and set up. The procedures of the paradigm allow the test fish to remain completely undisturbed by the experimenter for several days in their home (test) tank, eliminating human handling or human interference induced stress. We demonstrate that the development of cheap and simple automated home-tank-based learning paradigms for the zebrafish is feasible. We argue that such tasks will allow us to better characterize numerous cognitive and mnemonic features of the zebrafish, including elemental as well as configural learning and memory, which will, in turn, also enhance our ability to study neurobiological mechanisms underlying learning and memory using this model organism.

**Supplementary Information:**

The online version contains supplementary material available at 10.3758/s13428-023-02076-7.

## Introduction

Offering a reasonable compromise between biological complexity and practical simplicity, zebrafish is a promising model in neuroscience research (Gerlai, 2020). In addition to its simple but evolutionarily conserved brain (e.g., Mueller & Wullimann, 2009), zebrafish offer the possibility to utilize a variety of genetic tools allowing for exploration of molecular mechanisms underlying brain function and behaviour (Gerlai, 2020; Metscher & Ahlberg, 1999). With its nucleotide sequences highly homologous to those of human genes (Gerlai, 2020), the zebrafish is considered an excellent translational tool with a potential for being a model for a variety of human CNS disfunctions and underlying disease mechanisms (Kalueff et al., 2014; Stewart et al., 2014). Over the last decade, these qualities of the zebrafish have been mainly exploited in studies exploring toxicology (Dai et al., 2014), behavioural processes (Kalueff et al., 2013), pharmacology (Goldsmith, 2004) and cognitive abilities (Gerlai, 2020; Meshalkina et al., 2017).

In the domain of learning and memory, the zebrafish has also been well utilized. Adult zebrafish have been found to be able to exhibit the two main forms of associative learning seen in higher order vertebrates. They can make elemental association between a single stimulus (conditioned stimulus, or CS) and a reinforcer (unconditioned stimulus or US), let the latter be appetitive (rewarding) (Al-Imari & Gerlai, 2008; Colwill et al., 2005; Sison & Gerlai, 2010) or aversive (punishing) (Blank et al., 2009; Pather & Gerlai, 2009). Importantly, zebrafish have also been found to be able to acquire configural (relational) association among multiple different elements of their environment (multiple CSs) and a reinforcement, for example, during spatial learning (Karnik & Gerlai, 2012) or in delayed-matching-to-sample tests (Bloch et al., 2019). Potential mechanisms underlying associative learning abilities of adult zebrafish remain poorly understood. Even the methodological aspects of zebrafish learning tasks are in their infancy, as demonstrated by the orders of magnitude fewer publications found on this topic with zebrafish as compared to those on rodents.

In addition to the relative paucity of studies on cognitive and mnemonic characteristics of the zebrafish, existing learning protocols developed for this species have been found notoriously unreliable, yielding results that are often difficult to replicate compared to those obtained in studies with other species (see e.g., the proboscis extension reflex protocol in bees (Giurfa & Sandoz, 2012) or the Morris water maze in rodents (Morris, 1984; Vorhees & Williams, 2006)). Nevertheless, the number of zebrafish learning paradigms is rapidly increasing (see reviews by Kenney, 2020; and Gerlai, 2020b) allowing the quantification of the same or similar learning phenomena with the use of distinct methods. For instance, an inhibitory avoidance task using electric shock in a small aquarium with a black and white area allowed the investigators to achieve one-trial learning in zebrafish (Blank et al., 2009). A conceptually similar learning protocol with different settings was also successful (Lal et al., 2018). In this latter task, fish associated the side of the aquarium having a green light with electric shocks. Also, using this paradigm, Lal et al. (2018) were able to identify a subpopulation of neurons essential in this form of learning. Despite the sporadic successes, however, most learning tasks yield variable results. Given the large number of methodological differences among them, comparing the results they provide and ascertaining their validity has been often difficult.

Perhaps the most problematic methodological issue inherent in the great majority of learning tasks employed with zebrafish today is the involvement of human handling. In most learning tasks, the experimental zebrafish need to be transferred from their home tank to the experimental tank for training and testing. This transfer procedure includes aversive encounters with the human experimenter. Deleterious effects of human handling are well documented in the rodent literature. For example, the identity of the experimenter, e.g. the experimenter’s gender has been found to matter and can modify stress and anxiety in the rodent (Katsnelson, 2014; Sorge et al., 2014). Uncontrolled differences in handling, and associated factors, have been found to decrease reproducibility of behavioural results in genetic analyses of mice (Bohlen et al., 2014; Crabbe et al., 1999).Arguably, zebrafish should be even more sensitive to human handling than rodents, as the latter have been domesticated, i.e., have experienced intentional and unintentional artificial selection against stress and anxiety responses towards the human breeder/experimenter, whereas the former species has not. Furthermore, human handling in case of the zebrafish often involves net chasing and removal from the water, a highly stressful procedure leading to temporary hypoxia in the fish. For instance, when adult zebrafish were chased with a net for 4 min, and exposed to air for 1 min before having been placed in a test tank (a handling procedure that is not far from what is regularly employed in zebrafish research), cortisol levels were found to increase reaching a peak 30 min after the handling, and returned to baseline only after 4 hours past handling (Pavlidis et al., 2013). Cortisol, the mammalian readiness hormone, is a well-established stress indicator in zebrafish too (Ramsay et al., 2006). Thus, the above results clearly demonstrate the lasting stress inducing effects of handling on zebrafish. In accordance with the above, aquatic model organisms, including the zebrafish, have been found to be particularly affected by a variety of environmental factors influencing replicability and reproducibility of results (Gerlai, 2019; Lieggi et al., 2019), variability of handling procedures likely being one of them. Although novel handling methods may be developed that minimize stress and anxiety, the ultimate solution would be to avoid human handling in its entirety. One way to accomplish this is to develop automated, home-tank-based learning paradigms.

Attempts to develop automated tasks have been made (Ajuwon et al., 2022; Aoki et al., 2015; Babkiewicz et al., 2021; Brock et al., 2017; Doyle et al., 2017; Hu et al., 2021; Manabe et al., 2013; Mueller & Neuhauss, 2012). However, a common drawback of these paradigms has been that they are not easily accessible to the zebrafish behaviour researcher. One reason is hardware, the other is software: the paradigms utilize custom made aquariums, and running them requires advanced programming skills (Mueller & Neuhauss, 2012). Purchasing the full system is sometimes possible, but can be pricey (Brock et al., 2017). Importantly, even with these systems, interaction between zebrafish and the experimenter is not avoided, as training and tests are not conducted in the home tank of the fish (Aoki et al., 2015; Brock et al., 2017; Mueller & Neuhauss, 2012). Another limitation of the current literature on automated learning tasks with zebrafish is that although these methods allow complete control over the delivery of CS and US, the experiments using these methods often lack proper control groups (Aoki et al., 2015; Brock et al., 2017; Manabe et al., 2013; Mueller & Neuhauss, 2012).

In sum, we argue that there is room for improvement. That is, there is a need for development of an automated learning paradigm that eliminates/reduces human handling, a task that is accessible to the zebrafish behavioural scientist because of its simplicity and low cost. Here, we describe a simple semi-automated home-tank system that has been designed using low-cost materials and electronics easily found at any aquarium retailer or electronic store. We employ this system with appropriate controls to explore two forms of elemental associative learning of zebrafish, one in which the fish are required to associate a single CS with a US, referred to from here onward as “simple associative learning”, and another in which two CSs, CS1 and CS2, must be distinguished because only one of the CSs is paired with the US, a type of learning task referred to as “discrimination learning”.

## Materials

### Animals

Adult wild-type (WT) zebrafish (6-month-old females) raised in the University of Toronto Mississauga (UTM) zebrafish facility were used in these experiments. The parental generation of these fish was obtained from a local pet store (Big Al’s Aquarium Warehouse, Mississauga, ON, Canada). The rationale for using this population is that these fish are genetically variable (high heterozygosity ratio of their loci and high genetic heterogeneity, variance, in the population) and thus do not possess unique strain-specific features, but rather are likely to be representative of zebrafish at large. All experimental subjects were first housed in a multi-stage filtration stand-alone high-density rack system (ZD660, Aquaneering, San Diego, California) until 6 months of age. One week prior to the start the experiment, fish were transferred to the experimental room and housed in a 280-l glass tank with the same water parameters (see below) as in the rack system (*n* = 20 per tank). This tank was equipped with an external overhang filter (AQ-78266WM model 30-60, Aqua-Tech, USA). In both the main facility and the experimental room, fish were kept on a 14 h light: 10 h dark cycle (lights started to turn on at 6:30 am and slowly increased in brightness to maximum by 7:00 am. Lights started to turn off at 8:30 pm, with lights completely off by 9:00 pm). All tanks were checked daily and maintained within optimal water parameters (temperature = 27°C, pH = 6.8, salinity = 250 μS, NH_4_OH = 0 ppm, NO_2_− = 0 ppm, NO3- = 0 ppm). All fish were fed pellets (Zeigler, Tropical-fish micro pellets) twice a day starting at 3 months of age.

### Semi-automated home-tank

**Fig. 1:**
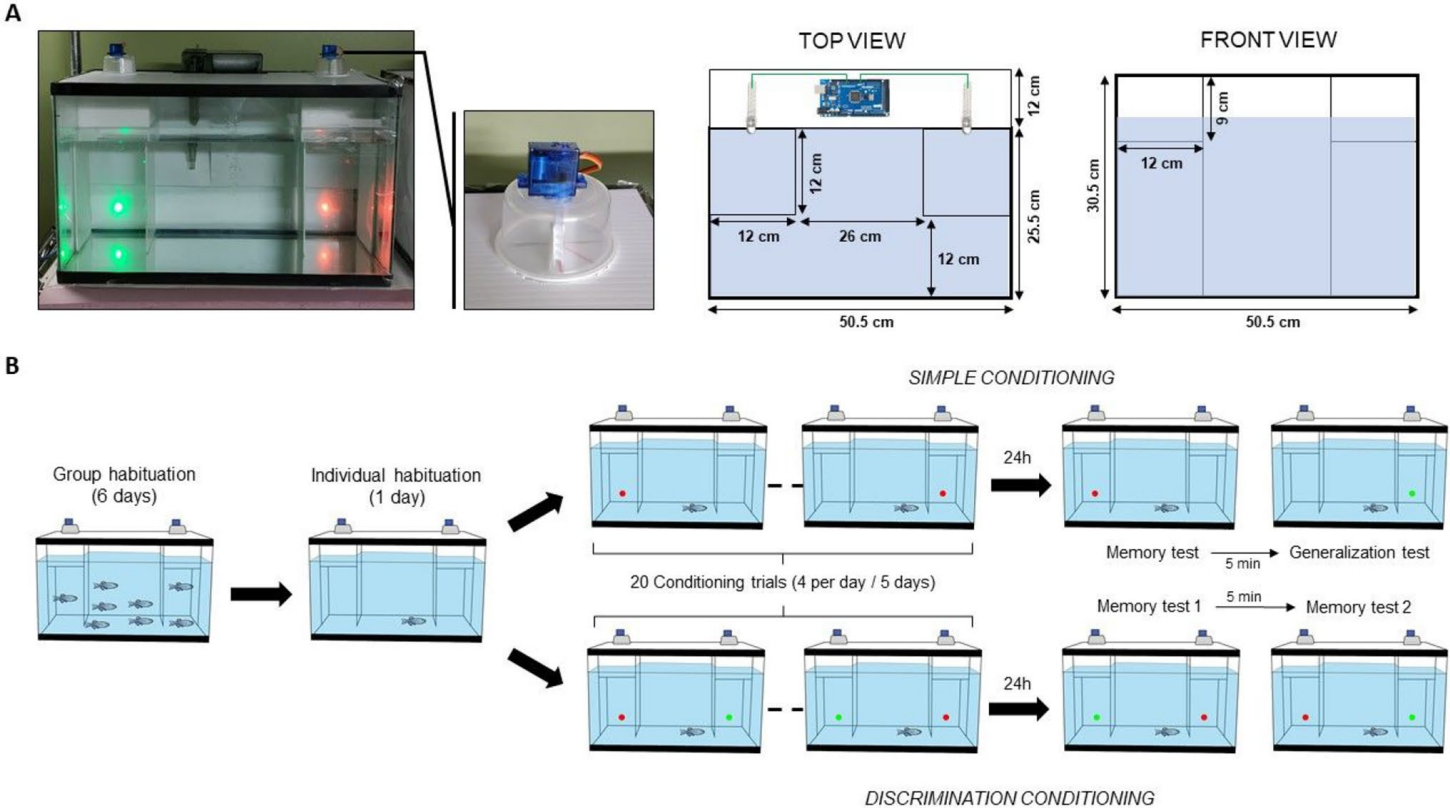
Automated home tank and experimental protocol. **A** The automated home tank consisted of a standard 40-l glass aquarium (50.5 cm x 30.5 cm x 25.5 cm). Two columns were added at the back corner of each tank using white PVC following the indicated dimension. A microcontroller was placed in a box at the back of the tank which controlled the RGB LED as well as the home-made feeder (which consisted of a rotating servomotor. A filtration system was placed on the back of the tanks to maintain optimal water quality during testing. **B** Two conditionings were used in this device. Fish were first habituated to the setup in groups of 5 for 6 days. Then, fish were transferred individually into one setup (five in parallel) and allowed to habituate for 24 h. Regardless of the protocol (simple or discrimination), fish received 20 conditioning trials over 5 days (four trials per day). For simple conditioning, the paired group received food in the column displaying the coloured-light (red in the example). In discrimination conditioning, the paired group received food in the column displaying the coloured-light that was chosen as CS+ prior to the experiment starting. For the unpaired group, food was released 30 min before/after the light display and pseudo-randomly in one of the two columns. This ensured unpairing the CS and US for this group. To avoid spatial association, rewarding light + food was administered equally in each column (pseudo-randomized across trials). Twenty-four hours after conditioning, memory was tested by presenting coloured lights used during conditioning without food reward

The semi-automated home-tank (Fig. 1A) was developed using easily accessible materials. We call it “semi”-automated, because as the description of procedures below shows, it still has a human handling component at the beginning and the end of each experimental day (Fig. S3). In addition, the protocols described in this manuscript represent classical conditioning, as they require CS-US association and not response-US association (operant conditioning) or multi-CSs relational learning (e.g. spatial learning). For example, the location of CS and US was irrelevant in our training methods. The experimental “home” tank of the paradigm was a 40-l glass tank (50.5 cm x 25.5 cm x 30.5 cm, Aqueon standard 10-gallon rectangular aquarium with black trim, obtained from Big Al’s Aquarium, Mississauga, Canada). The side, back and top of the tank was covered using white corrugated plastic sheets to prevent visual access to external environmental cues (183 cm x 91 cm x 0.3 cm, Home Depot, Mississauga, Canada). At the back corner of each tank, two columns were made of white PVC. In these two columns, visual stimuli were displayed using two RGB LED light, and food could be released from two homemade feeders (Fig. 1A, Fig. S1). These feeders were composed of a rotating servomotor (Micro servo 9G SG90, Miuzei, Japan) placed in a circular plastic container (#21470, Greenco, USA). The RGB LED light and homemade feeders were controlled via an Arduino type card (Fig. S2, S3; Elegoo Carte Mega 2560, Elegoo, China).

During each experiment, two coloured-lights were displayed: green (wavelength peak: 509 nm, brightness: 630 lux) and red (wavelength peak: 615, brightness: 580 lux). To prevent overfeeding and to keep fish motivated during the entire experiment, the home-made feeder released only three pellets per trial (0.5-mm community fish pellets, Northfin, Canada).

Three cameras (Raspberry Pi Camera Module 2, Raspberry, UK) controlled via a raspberry pi card (Raspberry pi 3B+, Raspberry, UK) were used to record the behaviour of the experimental zebrafish during the training trials and memory tests. One camera was used to record two automated tanks (Fig. S4). These cameras were controlled through WiFi with a computer placed outside of the experimental room. Thus, the training trials and memory tests were conducted without the stress-inducing presence of the experimenter.

### Habituation

Regardless of the conditioning protocol (simple or discrimination learning), 1 week prior to the conditioning, fish were placed into a habituation tank (*n* = 5 per tank) that was identical to the ones used in the experiment (Fig. 1B). For the first 6 days, fish were fed twice a day (morning and afternoon) with the experimental pellets (0.5 community, Northfin, Canada). The food was released each day once in the left column and once in the right one (pseudo randomly to ensure both the left and right columns fed similar number of times in the morning and the afternoon). Thus, fish experienced that food was released in the two columns. At the end of the sixth day, fish were individually transferred to the experimental automated home-tanks. We employed five automated home-tanks in parallel during training the fish. After having been transferred to their new home (and test) tanks (one fish per tank), fish were allowed to habituate to the tank for 24 h prior to the start of training, which was expected to reduce stress induced by human handling. After this 24-h period, the experiment started, and fish received either simple or discrimination conditioning, the training trials.

### Simple conditioning

During simple conditioning (Fig. 1B, Fig. S5), fish were trained to associate one coloured-light (red or green CS) with a reinforcement (food US, 3 pellets). The conditioning consisted of 20 trials distributed over 5 days (i.e., four trials per day) for both the paired and the unpaired group of fish. For the paired group, a coloured-light was displayed in one column for 60 s, and food was released after 45 s of light presentation in that exact same column (i.e., coloured-light and food presentations were overlapping for 15 s). Thus, fish were able to build an association between the CS and the US. To prevent association with other cues (e.g., spatial cues), light + food were presented in both the right and the left column twice per day pseudo-randomized according to the side. For the unpaired group (control), the coloured-light was also displayed for 60 s in one column, but food was released 30 min before or after the light display period and either in the same or opposite column. Thus, CS and US were unpaired, and acquiring associative memory was not possible. Similar to the paired group, timing of food administration (before or after light) and feeding column (left or right) were pseudo-randomized between fish. During conditioning, food pellets sank in the column as soon as they entered the water and they remained at the bottom of the column if the experimental fish did not eat them. Therefore, it was possible to determine when a fish was not eating the pellets, and to discard that fish from the experiment (48 fish were tested and ten were discarded).

Twenty-four hours after conditioning was completed, both paired and unpaired groups were tested for their memory and for what we call “generalization”. During the memory test, the coloured-light that was previously rewarded during conditioning (red or green) was displayed for 60 s in one column without any food reinforcement. The column was pseudo-randomly selected to have 50% of fish tested at right and 50% at left. During the generalization test, another coloured-light was displayed without food reinforcement (i.e., green if red was rewarded during conditioning and vice versa). Once again, column was pseudo-randomly selected. The two tests were separated by 5 min and the order was pseudo-randomly selected to balance the percentage of fish starting with each type of test.

### Visual discrimination conditioning

During discrimination conditioning (Fig. 1B, Fig. S5), fish were trained to differentiate two coloured-lights (red and green) according to their association (or not) with a food reinforcement (three pellets). The conditioning consisted of 20 trials distributed over 5 days (i.e., four trials per day) for both the paired and the unpaired group of fish. For the paired group, two coloured-lights were displayed (one in each column) for 60 s, and food was released after 45 s of light presentation in the column displaying the rewarded coloured-light (i.e., the coloured-light and food presentation periods were overlapping for 15 s). Thus, fish were able to build an association between the CS and the US (CS+ ➔ US; CS- ➔ no US). To prevent association with other cues (e.g., spatial cues), the rewarded light + food were presented in both columns twice per day in a pseudo-randomized order. For the unpaired group (control), the coloured-lights (CS1: green and CS2: red) were also displayed for 60 s, one in each column, but food was released 30 min before or after the light display in one of the columns (pseudo-randomized between trials). Thus, CS+ (or CS-) and US were unpaired, and acquiring associative memory was not possible. Similar to the paired group, timing of food administration (before or after light) and feeding column side (left or right) was pseudo-randomized between fish. During conditioning, food pellets sank in the column as soon as they entered the water and remained at the bottom of the column if the experimental fish did not eat them. Therefore, it was possible to determine when a fish was not eating the pellets, and to discard that fish from the experiment (40 fish were tested and four were discarded).

Twenty-four hours following the end of conditioning, fish of the paired and unpaired groups were tested for their memory, the memory test. During the first memory test, the coloured-lights were displayed for 60 s, one in each column, without any food reinforcement. During the second memory test, light location was reversed (i.e., if for test 1, red was at right and green was at left, then for test 2, red was presented at left and green at right). The two tests were separated by 5 min, and the order was pseudo-randomly selected to balance the percentage of fish starting with each type of test.

### Data analysis

For each experiment, the videos were analysed using BORIS software (Friard & Gamba, 2016) which allowed us to quantify the duration of time fish spent in each column. The quality of the video-recordings (Fig. S4), as well as the use of the coloured-light display made automatic tracking complex. The use of high-definition IR cameras combined with IR light will allow for precise automatic tracking throughout training and probe trials in future experiments. Nevertheless, BORIS is an accurate software that provided sufficiently high-quality data for our current study. During conditioning trials, data were collected for the first 45 s of coloured light presentation (prior to food reward administration) for the first trial of each day. Thus, we ensured that our results remained unaffected by any cues other (e.g. sound of motor, odour of food, vibration when food entered the water) than the coloured-light itself. For each memory test, this time was measured during the entire 60 s of coloured-light display.

For the simple conditioning experiment, the time spent in the column of the rewarded coloured-light during the 45 s of coloured light presentation was compared between paired and unpaired groups using a linear mixed model with fish ID considered as a random factor to account for the repeated measurement design. The model was built as follows: Time~Group*Trial*Colour + (1|Fish.ID). Since the colour parameter was not significant, it was removed from the model for the final analysis: Time~Group*Trial + (1|Fish.ID). During the memory and generalization test, time spent in the coloured-light column was compared between paired and unpaired group using a linear model built as follows using lm function: Time~Group*Colour. Once again, since colour was not significant, it was removed from the model: Time~Group.

For discrimination conditioning of paired group, the time spent in the column of the rewarded coloured-light (CS+) and unrewarded coloured-light (CS-) during the 45 s of coloured-light presentations were compared using a linear mixed model with fish ID considered as a random factor to account for the repeated measurement design. The model was built as follows: Time~CS*Trial*Colour + (1|Fish.ID). Since the colour parameter was not significant, it was removed from the model for the final analysis: Time~CS*Trial + (1|Fish.ID). The time spent in each CS column during memory test 1 and 2 was compared using a linear mixed model with fish ID considered as a random factor. The model was built as follows: Time~CS*Test*Colour + (1|Fish.ID). Neither the test, nor the colour parameters were significant. Consequently, test 1 and 2 have been independently analysed using a linear model: Time~CS.

Since neither CS1 (green) nor CS2 (red) were rewarded during the conditioning of unpaired group, the colour parameter was not considered in the analysis. For this reason, for conditioning trials, time spent in CS1 and CS2 column was compared using a linear mixed model with fish ID as a random factor: Time~CS*Trial. The performance in the two-memory tests was compared using a linear mixed model with fish ID as a random factor too: Time~CS*Test. The test parameter was not significant, so both tests were analysed independently using a linear model: Time~CS.

All the analyses were performed on R (R Core Team, 2021). Graphics were made on R utilizing the library ggplot2. The linear mixed models were performed using the function lmer() from the library lme4. The linear models were performed using the function lm(). The quality of each model was double checked using the function simulateResiduals() and testDispersion() from the package DHARMa. When data were not fitting a normal distribution, it was transformed following a Johnson transformation (based on the method of the percentiles) using RE.Johnson() function from the library Johnson. This last method was applied for the conditioning analysis of every experiment, as well as for the memory tests of the unpaired group in the discrimination conditioning experiment.

## Results

### Simple conditioning

During conditioning, whether red or green was used as CS did not significantly affect the fish’s performance (Colour effect: Χ^2^ = 0.73, *df* = 1, *p* = 0.39; Colour:Trial effect: Χ^2^ = 3.32, *df* = 1, *p* = 0.07; Group:Trial:Colour effect: Χ^2^ = 5.27, *df* = 4, *p* = 0.26). Similarly, whether red or green was rewarded during conditioning had no significant effect on the performance of fish during the memory test (Colour effect: *F* = 0.73, *df* = 1, *p* = 0.4; Group:Colour effect: *F* = 0.11, *df* = 1, *p* = 0.74) and the generalization test (Colour effect: *F* = 2.26, *df* = 1, *p* = 0.14; Group:Colour effect: *F* = 2.28, *df* = 1, *p* = 0.14). For this reason, data were pooled for colour in this experiment. Nevertheless, performance of fish for each coloured-light is presented separately in the supplementary material (Fig. S3).

**Fig. 2:**
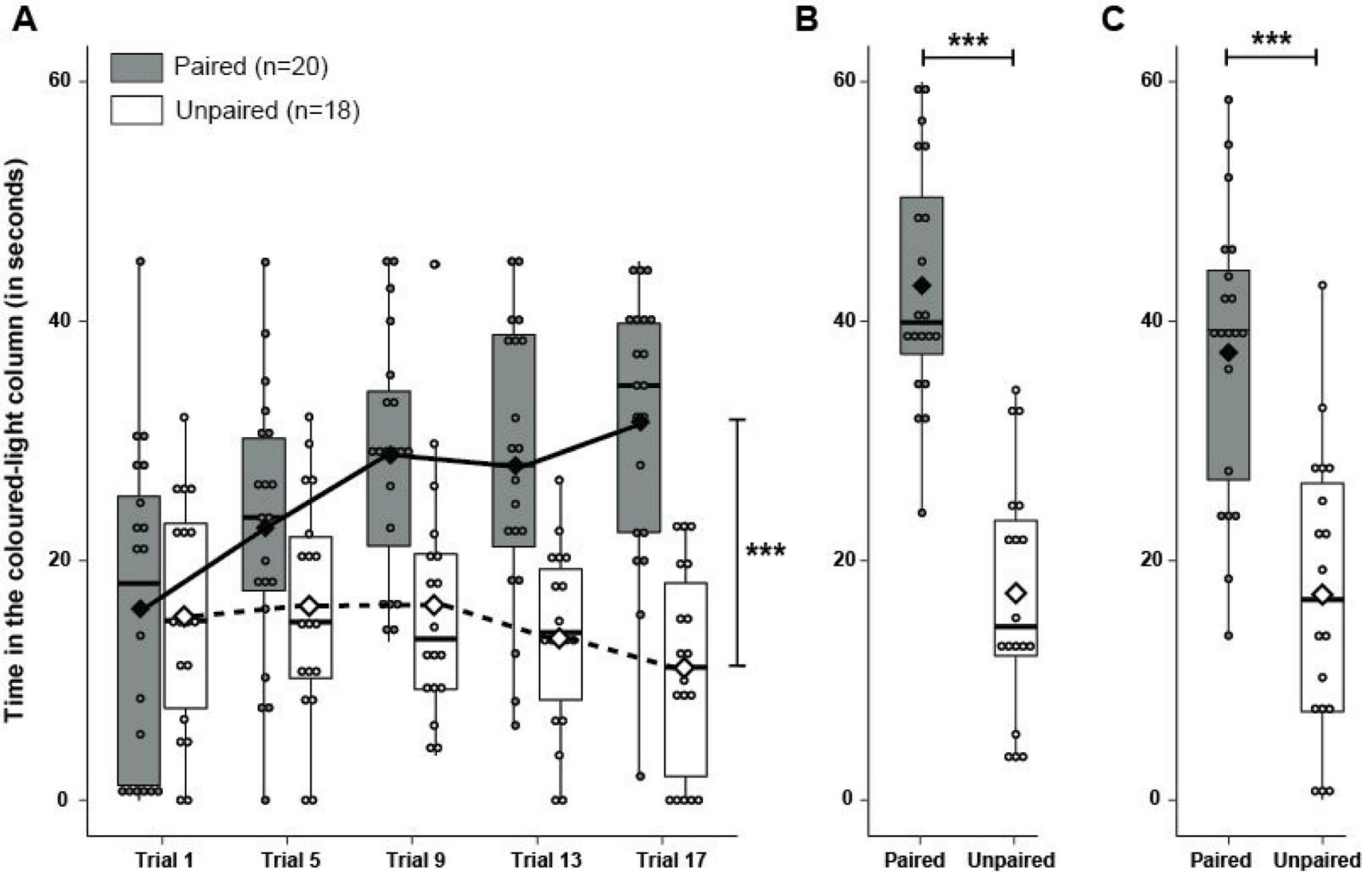
Simple conditioning. **A** Time (in seconds, median and quartiles) spent by fish from paired group (*n* = 20, *grey*) and unpaired group (*n* = 18, *white*) in the coloured-light column during the first 45 s of coloured-light display of the first conditioning trial of each conditioning day. **B** Time (in seconds, median and quartiles) spent by fish from paired group (*n* = 20, *grey*) and unpaired group (*n* = 18, *white*) in the coloured-light column during the 60 s of the memory test. **C** Time (in seconds, median and quartiles) spent by fish from paired group (*n* = 20, grey) and unpaired group (*n* = 18, white) in the coloured-light column during the 60 s of the generalization test. **A, B, C** *Small dots* represent individual data points, and the *diamonds* connecting the curve represent the mean of time for each group. ***p < 0.001

Fish from the paired group and the unpaired group significantly differed from each other regarding the trajectory of time spent in the coloured-light area across the conditioning trials (Fig. 2A, Table 1A). Briefly, while fish from the paired group increased the time spent in the coloured-light column across the trials (even before the reward was presented at each trial) as the training progressed, fish from the unpaired group decreased it.

**Table 1:**
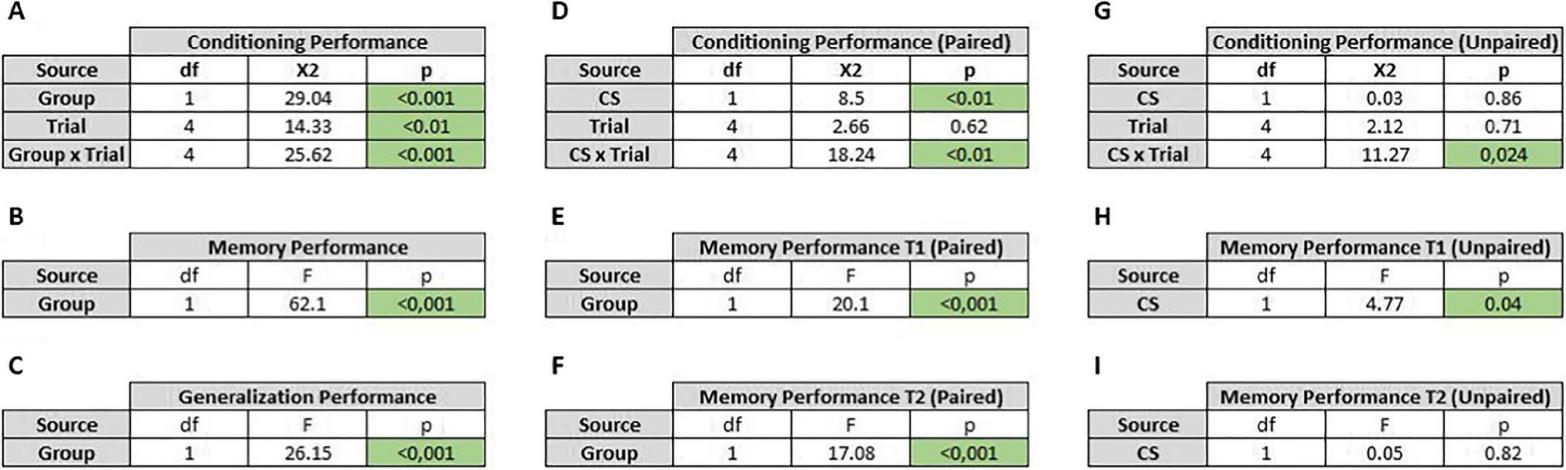
Summary of linear mixed model and linear model results. **A** Linear mixed model results for the performance of fish during simple classical associative (CS-US) conditioning (Fig. 2A). **B** Linear model results for the performance of fish during memory test (Fig. 2B). **C** Linear model results for the performance of fish during “generalization” test (Fig. 2C). **D** Linear mixed model results for the performance of fish (paired group) during discrimination conditioning (Fig. 3A). **E** Linear model results for the performance of fish (paired group) during memory test 1 (Fig. 3B). **F** Linear model results for the performance of fish (paired group) during memory test 2 (Fig. 3C). **G** Linear mixed model results for the performance of fish (unpaired group) during discrimination conditioning (Fig. 3D). **E** Linear model results for the performance of fish (unpaired group) during memory test 1 (Fig. 3E). **F** Linear model results for the performance of fish (unpaired group) during memory test 2 (Fig. 3F). Significant results are highlighted in green

During the memory test, fish from the paired group spent significantly more time in the coloured-light column than fish from the unpaired group did (Fig. 2B, Table 1B). Similarly, when tested for generalization with a new coloured-light, fish from the paired group also spent significantly more time than the unpaired group in the coloured-light column (Fig. 2C, Table 1C).

### Discrimination conditioning

Whether the red or the green light was rewarded did not have a significant effect on the fish’s performance during conditioning in the paired group (Colour effect: Χ^2^ = 0.28, *df* = 1, *p* = 0.59; Colour:Trial effect: Χ^2^ = 0.71, *df* = 1, *p* = 0.95; Colour:CS:Trial effect: Χ^2^ = 1.73, *df* = 4, *p* = 0.79). Moreover, during the memory test, the performance of the paired group also did not significantly differ according to which colour was paired with reward during conditioning (Test effect: Χ^2^ = 0.03, *df* = 1, *p* = 0.87; Colour effect: Χ^2^ = 0.15, *df* = 1, *p* = 0.69 Test:CS effect: Χ^2^ = 0.97, *df* = 1, *p* = 0.32; Colour:CS effect: Χ^2^ = 0.02, *df* = 1, *p* = 0.9; Test:Colour effect: Χ^2^ = 0.14, *df* = 1, *p* = 0.71; Test:Colour:CS effect: Χ^2^ = 1.69, *df* = 1, *p* = 0.19). For this reason, paired group data were pooled for colour for the graphs and subsequent data analysis. Nevertheless, results are also shown separated according to rewarded colour in the supplementary materials for further perusal (Fig. S4).

**Fig 3:**
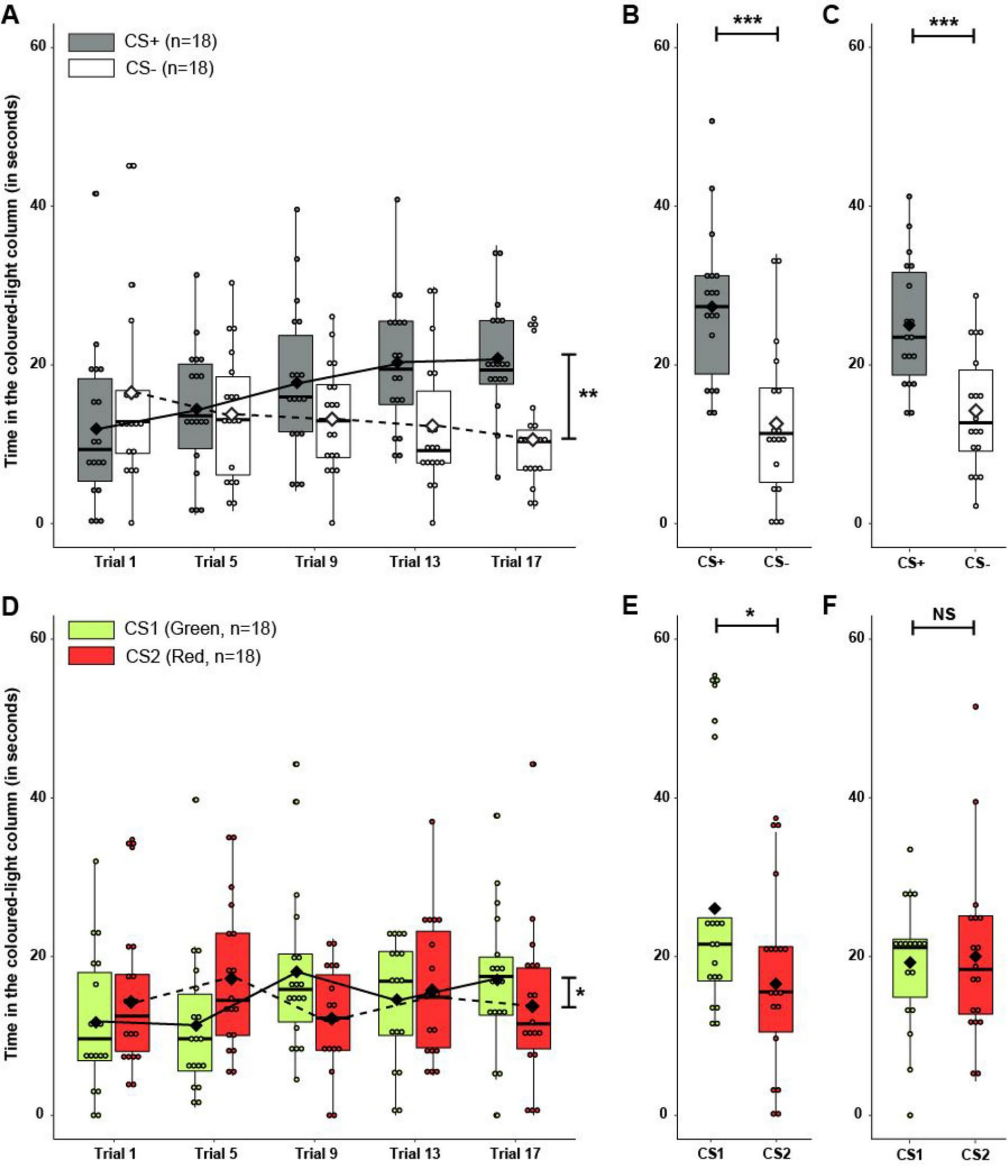
Discrimination conditioning. **A** Time (in seconds, median and quartiles) spent by fish from paired group (*n* = 18) in the CS+ (*grey*) and CS- (*white*) columns during the 45 s preceding the food reward release during the first conditioning trial of each conditioning day. **B** Time (in seconds, median and quartiles) spent by fish from paired group (*n* = 18) in the CS+ (*grey*) and CS- (*white*) columns during the 60 s of the first memory test. **C** Time (in seconds, median and quartiles) spent by fish from paired group (*n* = 18, *grey*) in the CS+ (*grey*) and CS- (*white*) columns during the 60 s of the second memory test. **D** Time (in seconds, median and quartiles) spent by fish from unpaired group (*n* = 18) in the CS1 (*green*) and CS2 (*red*) columns during the first 45 s of coloured-light display of the first conditioning trial of each conditioning day. **E** Time (in seconds, median and quartiles) spent by fish from unpaired group (*n* = 18) in the CS1 (*green*) and CS2 (*red*) columns during the 60 s of the first memory test. **F** Time (in seconds, median and quartiles) spent by fish from unpaired group (*n* = 18) in the CS1 (*green*) and CS2 (*red*) columns during the 60 s of the second memory test. **A, B, C, D, E, F** *Small dots* represent individual data points, and *diamonds* connecting the curve represent the mean of time for each group. *p < 0.05; **p < 0.01, ***p < 0.001; *NS* non-significant

During conditioning, fish from the paired group significantly increased their time spent in the CS+ column across the trials as the training progressed, and consequently decreased the time spent in the CS- column (Fig. 3A, Table 1D). Although the time spent in each CS column did not significantly differ for the unpaired group, the performance of these fish significantly varied across training trials (Fig. 3D, Table 1G).

Fish of the paired group spent significantly more time in the CS+ column than in the CS- during both memory test 1 (Fig. 3B, Table 1E) and test 2 (Fig. 3C, Table 1F). Similarly to the paired group, the performance of unpaired fish did not significantly change between the two memory tests (Test effect: Χ^2^ = 0.05, *df* = 1, *p* = 0.81; Test:CS effect: F = 2.4, *df* = 1, *p* = 0.12). However, when analysing each test independently, fish from the unpaired group spent significantly more time in the CS1 (green) column than in the CS2 (red) one during test 1 (Fig. 3E, Table 1H), but this difference was absent in test 2 (Fig. 3F, Table 1I).

## Discussion

In this study, we presented the design of a semi-automated-home tank apparatus, as well as procedures with which individual adult zebrafish could be tested for acquisition of associative memory using simple CS-US conditioning or discrimination conditioning with coloured-light as CS and food as US. The task is semi-automated in the sense that it starts with human handling, moving the experimental zebrafish to its new “home”. However, subsequent to this transfer, the experimental fish remains completely undisturbed as all stimuli, conditioned and unconditioned, are delivered using remote control, and the behaviour of the fish is recorded using cameras controlled from remotely, i.e., in the physical absence of the experimenter. Our results unequivocally demonstrated that zebrafish could learn in both types of conditioning tasks, simple learning and discrimination learning. Finding zebrafish to be able to acquire elemental associative memory is not surprising as similar results have been obtained before (Al-Imari & Gerlai, 2008; Colwill et al., 2005; Sison & Gerlai, 2010). Some of these learning studies employed automated tanks (Aoki et al., 2015; Brock et al., 2017; Doyle et al., 2017; Manabe et al., 2013; Mueller & Neuhauss, 2012). However, several of the prior learning studies conducted with zebrafish had limitations, which we argue our current study has now addressed.

For example, in some previously published studies only a paired group of zebrafish was tested, and an appropriate control group, e.g., for which the CS and US were unpaired, was absent. In our current study, two independent groups of fish were tested in parallel: a paired and an unpaired for both the simple as well as for the discrimination conditioning procedures. Unpairing the CS and US as a control training condition was introduced in 1943 (Harris, 1943). It is an excellent control for the paired group as fish in both groups receive the same number of training trials and the same stimuli (both the CS and the US) under identical conditions (except the pairing). Thus, the performance characteristics of training are identical for the two groups (same motor, perceptual and motivational requirements), but CS is predictive of the US for the paired group and not predictive for the unpaired group. Under some circumstances unpairing may not be fully possible, and depending on how training trials are run, animals in the unpaired group could acquire memory of certain aspects of the task (Schleyer et al., 2018). Nevertheless, our current results suggest that zebrafish of the unpaired group did not acquire memory of any association between the CS and US we presented to them during training, regardless of whether we employed the simple or the discrimination training procedure. Furthermore, in the discrimination experiment, unpairing the CSs and the US allowed us to determine any potential innate coloured-light preference of zebrafish.

Zebrafish colour preference has been extensively studied over the past years (Avdesh et al., 2012; Buatois et al., 2021; Spence & Smith, 2008). The general conclusion of these studies has been that zebrafish possess innate colour preference, although what actual colour is preferred by zebrafish has been controversial and appeared to have depended upon methodological aspects of the studies. As innate colour preferences can confound the results of any learning study that uses visual cues, one must always explore and consider them. Our results show that at the beginning of the discrimination learning experiment, no preference for the green or red light was evident, confirming prior findings in which an RGB LED panel was employed to test coloured-light preference in zebrafish (Buatois et al., 2021). Nevertheless, our results also suggest that as training progressed, zebrafish from the unpaired group developed a modest preference for the green light. We note that the green light we employed was slightly brighter than the red one (630 lux vs 580 lux, respectively). Preference for stronger illumination has been shown in zebrafish, but is debated (Facciol et al., 2017; Gerlai, 2020; Maximino et al., 2012). Thus, whether it was the wavelength of the employed green light or its intensity that zebrafish preferred in the current study will need to be investigated in the future. Whether the CS was the red or the green light, however, made no difference in both the simple and discrimination conditioning, i.e., the modest colour bias we detected in the unpaired group did not bias learning performance in the paired groups.

Perhaps the most important aspect of our study is that our newly developed apparatus and procedure allowed us to conduct habituation, training trials and memory probe trials with zebrafish without having any human intervention, i.e., the experimental fish did not have to be handled or interacted with for the entire period of the multi-day experiment. All stimuli were delivered in an automated manner, and the behaviour of fish was monitored and recorded also in the absence of an experimenter in the testing room. In most learning studies conducted to-date with zebrafish this has not been accomplished. In these studies, interaction between the experimenter and the fish can occur at a variety of stages of the experiment. Even when conducted with utmost care, interaction between the fish and the experimenter can have robust fear inducing effects. For example, most learning tasks require multiple training trials, i.e., removal of the test fish from their home tank, their transfer to the training/test tank, removal from the test tank and transfer back to the home tank, a process that is repeated several times. This is an invasive procedure that often leads to sensitization to human handling, i.e., enhanced fear responses, including freezing. Even if the fish are kept in the test tank for the entire duration of training trials and memory tests, stimulus delivery often requires the experimenter moving around the tank, pulling levers/pulleys, placing food into the tank, turning on switches, adjusting computers. Similarly, most often, the experimental fish must be manually fed during the experiment. Large moving objects (e.g. the human experimenter) approaching or just visible from the test tank will induce robust fear reactions in this small prey fish, effectively negating the relevance of experimenter provided CS and US. Alternatively, the fish may associate the experimenter’s hand with food reinforcement, interfering with CS - US association. In our current paradigm, none of this happened. The lack of experimenter induced interference, we argue, is a major step forward in zebrafish cognition research.

Learning has been explored in zebrafish using different protocols such as elemental conditioning using social reward (Sison & Gerlai, 2010), discrimination learning using visual (Colwill et al., 2005) or olfactory stimuli (Namekawa et al., 2018) and configural/relational methods such as reversal conditioning (Colwill et al., 2005; Kuroda et al., 2017), spatial conditioning (Karnik & Gerlai, 2012) or delayed-matching to sample (Bloch et al., 2019). Although the range of methods employed clearly demonstrated good learning and memory performance of zebrafish, the number of methodological differences make the results of these studies difficult to compare. Also importantly, the lack of consistency in performance features of these paradigms, e.g. their motor, perceptual and motivational demands, and the varied parameters of methods employed to achieve acquisition, consolidation, and the different ways recall of the acquired associative memory is tested, all represent complex problems for the molecular neurobiologist interested in using these methods to uncover mechanisms underlying learning and memory. Thanks to its simplicity and expandability, our system may address this issue. Here, we presented two classical conditioning protocols, a simple associative learning task and a discrimination associative learning task. Our classical conditioning protocols did not consider the position of the experimental zebrafish. One may argue that the location of the fish at the moment of stimulus delivery may affect the time the fish spends on particular sides during the conditioning trials. However, the pseudo-randomization of stimulus display sides, as well as the use of the exact same procedure employed for fish of the paired and unpaired groups ensures the minimization of any spatial bias. We also note, however, that upgrading the system to an operant conditioning task would be possible by adding a motion sensor (or video-tracking based fish detection) in each column for instance. Regardless of the way one may uses our new device (classical or operant), it would also be possible to adapt it for matching-to-sample, non-matching-to-sample or higher order conditioning protocols using multiple LEDs with different colours. Similarly, configural/spatial learning can also be tested by placing LEDs at distinct locations in the columns. Even motivation may be analysed by controlling the location and amount of food delivered using progressive interval or ratio reinforcement schedules, all possible due to the simple programmability of the system and the full control of stimulus delivery it allows.

We also emphasize the efficiency of the system, i.e., the fast development of acquisition of memory it was able to achieve in zebrafish. For example, Colwill et al. (2005) needed 64 trials to achieve significant acquisition in zebrafish. Our protocol employed only 20 trials. It is also important to note that our trials spanned several days and led to consistent improvement of learning performance over time. We consider this an achievement because zebrafish are known to be difficult to motivate with food as the fish usually satiate fast (Daggett et al., 2019). However, as our results demonstrate, satiation with food (i.e., devaluation of the US) was not an issue in our paradigm where the intertrial intervals during training could be made long enough in the home-tank. Whether food satiety is going to be a limitation in future configural learning protocols that may require larger number of, and/or more frequent, conditioning trials will need to be empirically tested. We note that in our current protocols, three food pellets were released for each trial, but it is possible to decrease this quantity and thus to increase the number of trials per day (up to eight trials with the current version of the feeder) without satiating the fish to food. Moreover, since the device is a home-tank that completely removes the necessity to handle the fish, it is also simple to increase the number of conditioning days without inducing stress or anxiety.

Our results also allow us to hypothesize about what actually zebrafish learned in our tasks. For example, memory performance of the zebrafish after simple conditioning suggests that the CS the fish associated with the US was the presence of light irrespective of what colour it had. This conclusion is supported by finding good response in the “generalization” memory probe to the coloured-light to which the fish were naïve, i.e. were never trained with during the acquisition trials. Such a generalization has not been tested or described in zebrafish. Our findings also demonstrate that this “generalization” is not because zebrafish cannot distinguish the two coloured lights (red and green) employed. The memory probe trials conducted after the colour discrimination conditioning demonstrated that experimental zebrafish could clearly distinguish and remember CS+ and CS-. Thus, the most likely explanation for the observed “generalization” in the elemental conditioning experiment is that in terms of saliency of features of a light stimulus, zebrafish paid more attention to presence versus absence of light, and only when the difference between the colours was made informative did they attend to this aspect of the stimulus. Admittedly, however, our knowledge on how zebrafish interpret coloured-light in a variety of behavioural contexts is preliminary. Our current system will facilitate investigation in this research domain too. For example, it allows controlling brightness and colour, as well as contrasting and comparing these features of CSs during training and/or memory tests.

One of the system’s unique features is that in it fish are individually tested for several days, and therefore are isolated from conspecifics during the entire period. In addition to eliminating human interference, this is a main advantage as it also allows the experimenter to follow temporal changes in acquisition of CS-US association for every subject without having to individually mark the subjects and without the potentially complex confounding effects of social interaction during learning (e.g., Doyle et al., 2017). However, some may argue that isolating zebrafish for several days is also a concern given the social nature of zebrafish (Miller & Gerlai, 2011) and the potential deleterious (stressful) effects of social isolation. However, recent findings have shown that social isolation can actually reduce stress responses in zebrafish, including behavioural signs of fear and anxiety as well as level of cortisol (Parker et al., 2012; Shams et al., 2015). This is likely because crowding zebrafish in standard small high-density tanks, as is customary in most zebrafish facilities, may be actually stressful for zebrafish. In accordance with this argument, we note that all of our experimental subjects in our paired groups exhibited good learning performance, and none of the experimental fish (paired or unpaired, elemental association or discrimination trained) exhibited any signs of anxiety, fear, or stress. For example, none of our fish showed freezing, a natural species-specific behavioural response to aversive stimuli and contexts in zebrafish and a major issue in other learning tasks conducted in the past (Gerlai, 2013).

Last, we note that the LEDs allowing us to deliver CSs in our current hardware design employ the same RGB system that computer monitors or handheld devices, like tablets and notepads, use. For a small amount of extra cost, thus, one could easily expand/upgrade our system with RGB LED panels that would allow for displaying coloured shapes, the use of which has been gaining increasing popularity in zebrafish (Gerlai, 2017) as well as in other animal behaviour research applications (Chouinard-Thuly et al., 2017). Computer monitors have also been successfully employed to deliver social cues, e.g. animated images of zebrafish (Gerlai, 2014, 2017), and such cues have been shown to serve as excellent reinforcers in appetitive learning tasks developed for the zebrafish (Al-Imari & Gerlai, 2008). Thus, the experimenter would have a range of tools and options to increase the complexity of the learning and memory paradigm.

Finally, one of the main limitations linked to the different automated systems employed in the past is the difficulty to afford them, either because the system is unique (Mueller & Neuhauss, 2012), or because it has expensive components mandatory to purchase (Brock et al., 2017). This can make replication by other scientists working on zebrafish difficult. However, the automated home tank described in this paper has been developed using cheap materials, common tanks and accessible electronics/software (Table. S1) allowing zebrafish researchers to replicate the system for their laboratories.

The next topic we wish to discuss is the future expansion, further development, of our hardware/software design and procedures. We see two fundamental future directions. One, development of an animal behaviour-based feedback loop. That is, making the delivery of stimuli (US primarily but in some applications CSs too), contingent upon certain responses (e.g., location or particular movement) of the zebrafish. Such operant procedures (and hardware/software solutions) would significantly expand the flexibility/utility of our system. The second major direction, we foresee, is to develop methods (and hardware solutions) that would allow the use of olfactory cues as well as water soluble agents. Especially the latter is crucial if one wants to utilize home-tank-based systems for drug (small molecule) screens. As mentioned before, our current system utilizes a recirculating filtration method. The next step that would allow the use of water-soluble agents is a flow-through system that would incorporate the administration as well as the removal of these compounds from the home tank.

In summary, here we presented the design of a simple and cheap system that allowed us to train zebrafish and test their memory without any human interference. The system monitored the behaviour of individual zebrafish for 7 days isolated in what we call their home-tank. During this period, no sign of stress/anxiety was observed, and the fish remained active and were able to acquire associative memory both when the learning task required making an elemental association (between a single CS and US) and when it required discrimination between CS+ (the US-reinforced CS) and CS- (an unreinforced CS). We hope that the lack of confounding effects of human interference afforded by this method, along with its simplicity, expandability and low cost, will make it popular and will help those who would like to study the psychological and neurobiological mechanisms of learning and memory in this genetically tractable and translationally relevant species.

### Supplementary information

ESM 1 (DOCX 3.15 kb)
