# Supplementary material for: A simple semi-automated home-tank method and procedure to explore classical associative learning in adult zebrafish
Source: Behav Res Methods. 2023 Feb 22;56(2):736–49. doi: 10.3758/s13428-023-02076-7 (PMC10830691; doi:10.3758/s13428-023-02076-7)
Supplement: Supplementary file 1 — (DOCX 3.15 kb) [file 13428_2023_2076_MOESM1_ESM.docx]

**A simple semi-automated home-tank method and procedure to explore classical associative learning in adult zebrafish**

Alexis Buatois^1^, Zahra Siddiqi^1^, Sadia Naim^1^, Tulip Marawi^1^, Robert Gerlai^1,2^

^1^ University of Toronto Mississauga, Department of Psychology, 3359 Mississauga Road, Mississauga, Ontario, L5L 1C6, Canada

^2^ University of Toronto, Department of Cell and Systems Biology, 25 Harbord St, Toronto, Ontario, M5S 3G5, Canada

**Corresponding authors**:

Dr. Robert Gerlai and Dr. Alexis Buatois

**Address correspondence:**

Robert Gerlai, PhD

Department of Biology

University of Toronto Mississauga

3359 Mississauga Road

L5L 1C6 Mississauga, Ontario, Canada

**Mails:**[robert_gerlai@yahoo.com](mailto:robert_gerlai@yahoo.com); [buatoisalexis@gmail.com](mailto:buatoisalexis@gmail.com)


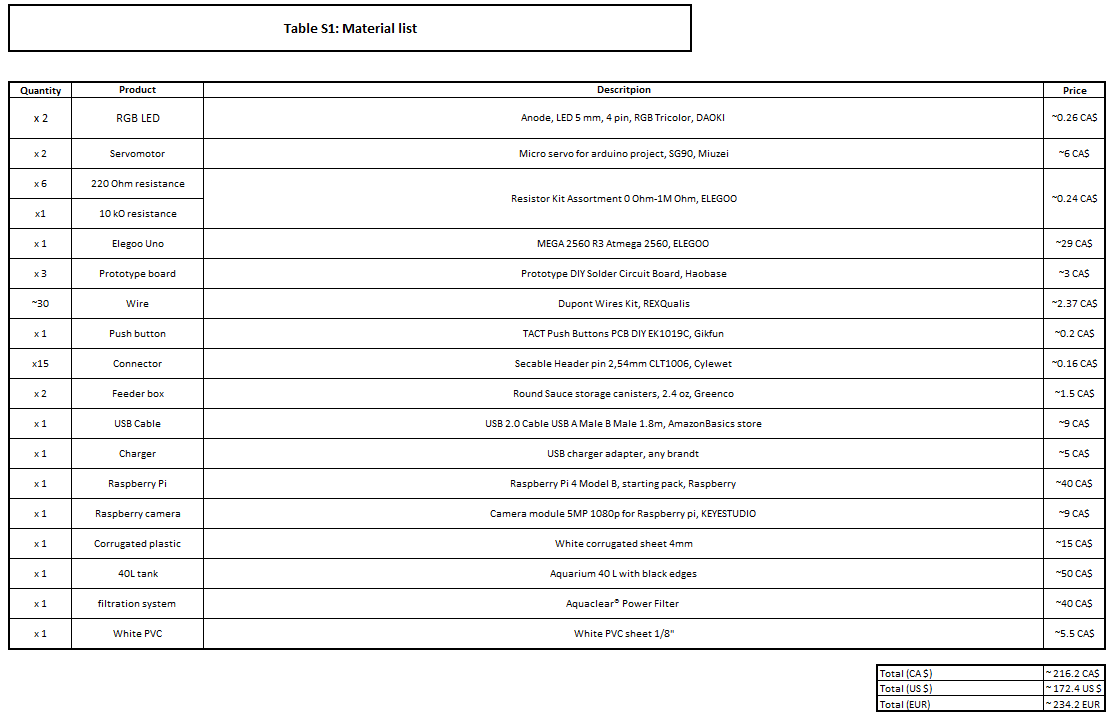


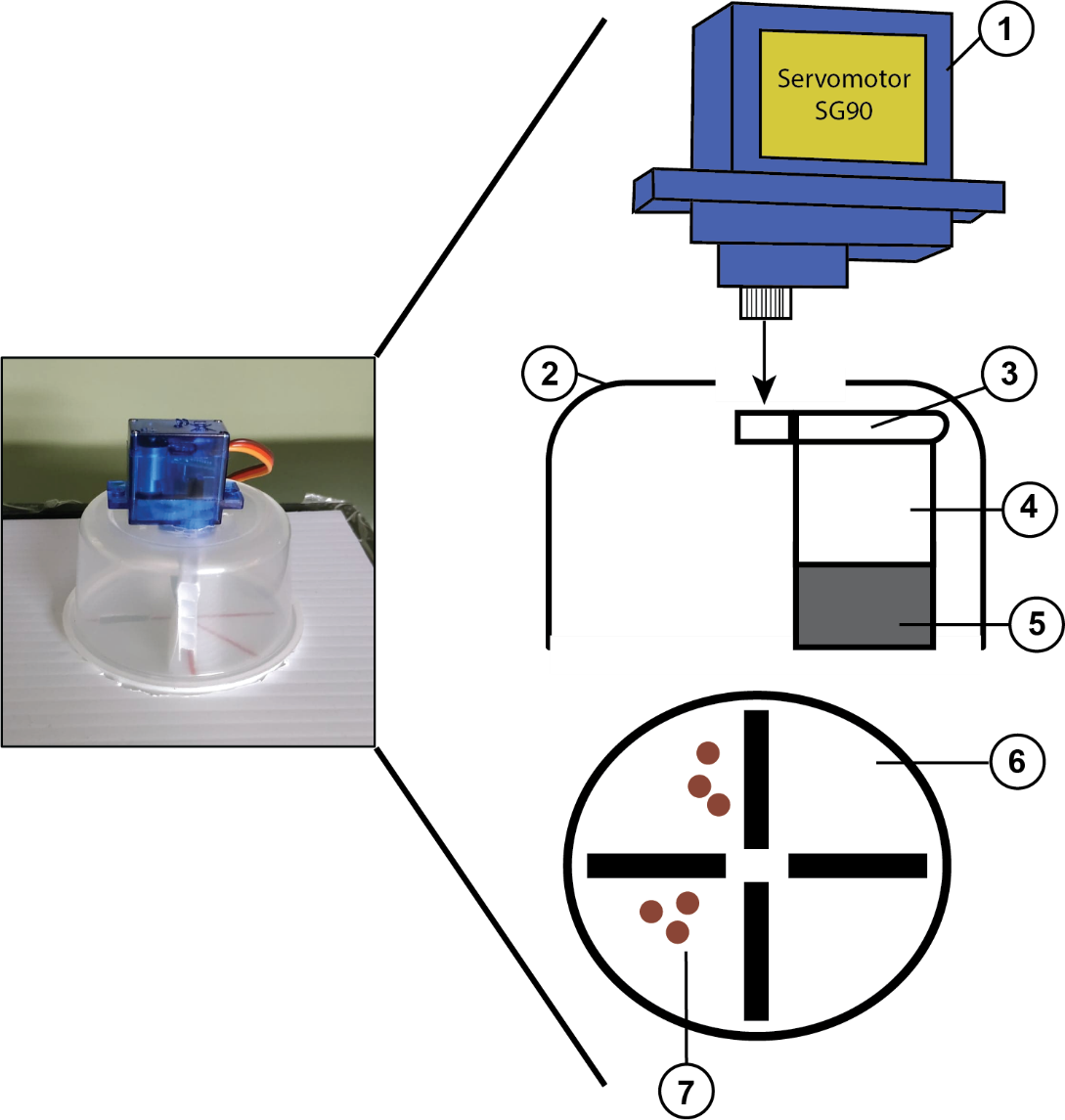


**Figure S1. Exploded view of the home-made feeder used with the semi-automatic home tank.** The head of a servomotor **(1)** is positioned at the center of the bottom part of a plastic container **(2)** in a hole manually cut at the dimension to fit. Servomotors are always coming with a propeller **(3)** that can be placed on the head. This propeller has been customized by passing it in a little piece of corrugated plastic **(4)** to have a rigid structure on which a piece of plastic sheet **(5)** has been glued. This piece was acting as a groom that push the pellets. Dimension of this piece could differ according to the plastic container you are using. It is important that the plastic sheet is long enough to be a bit curved on the lid once closed to facilitate pushing. The lid **(6)** of the plastic container has also been modified by adding four slots (black lines) every 90°. The 3 pellets **(7)** are thus pushed by the groom in the next slot. Thus, it is possible to use one feeder four times per day, and with the quantity of food needed for your experiment. It is also possible to design the slot larger to use another type of food, or to make more slot if the wish is to feed more times. The pellets are placed on **(6)** every morning before conditioning and then the feeder is closed and placed on the system.


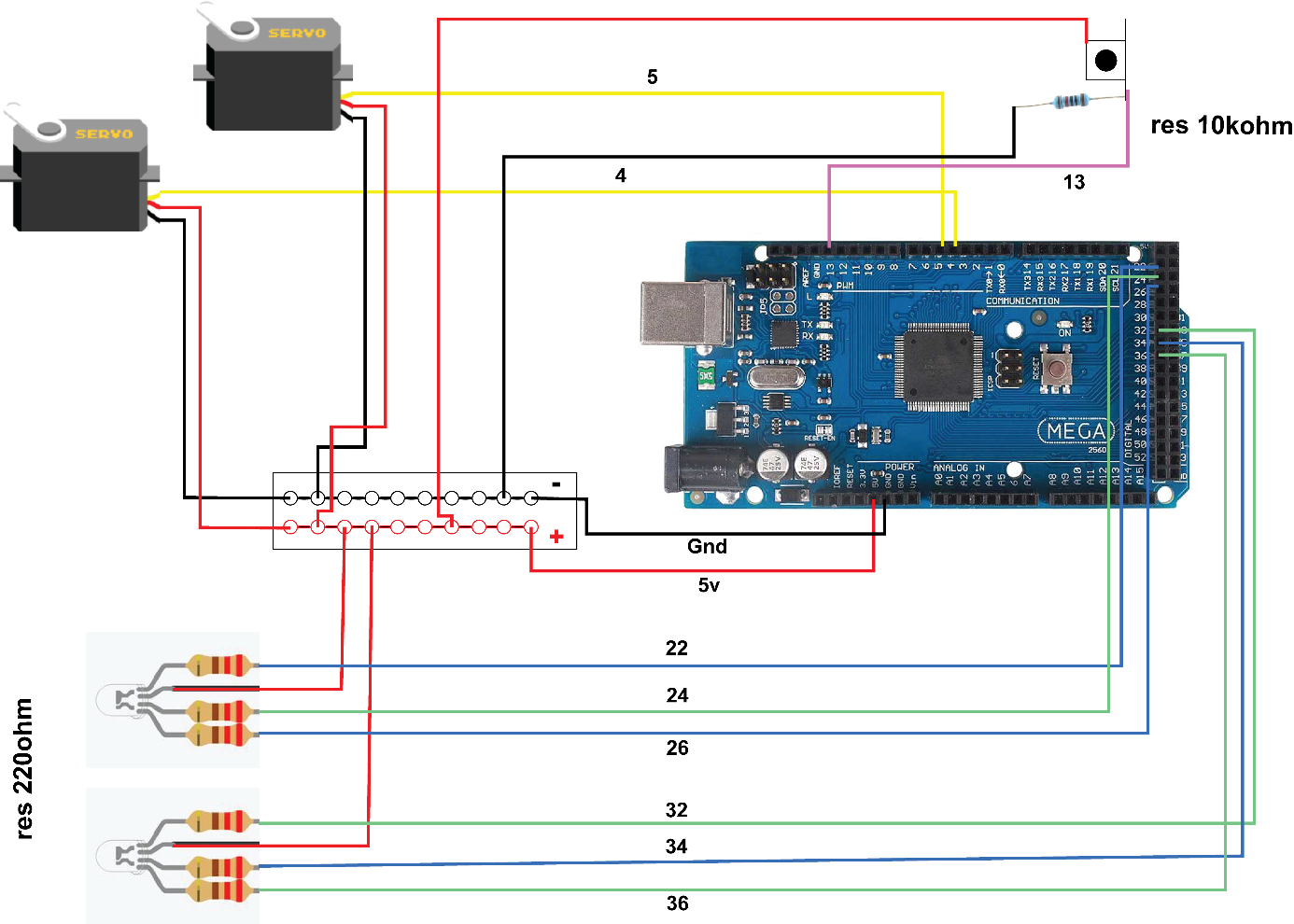


**Figure S2. Electronic plan of the system.** Two RGB LEDs, two servomotors and one push button were connected to an Arduino type acquisition card thanks to electronic wires following this plug-in plan.

**
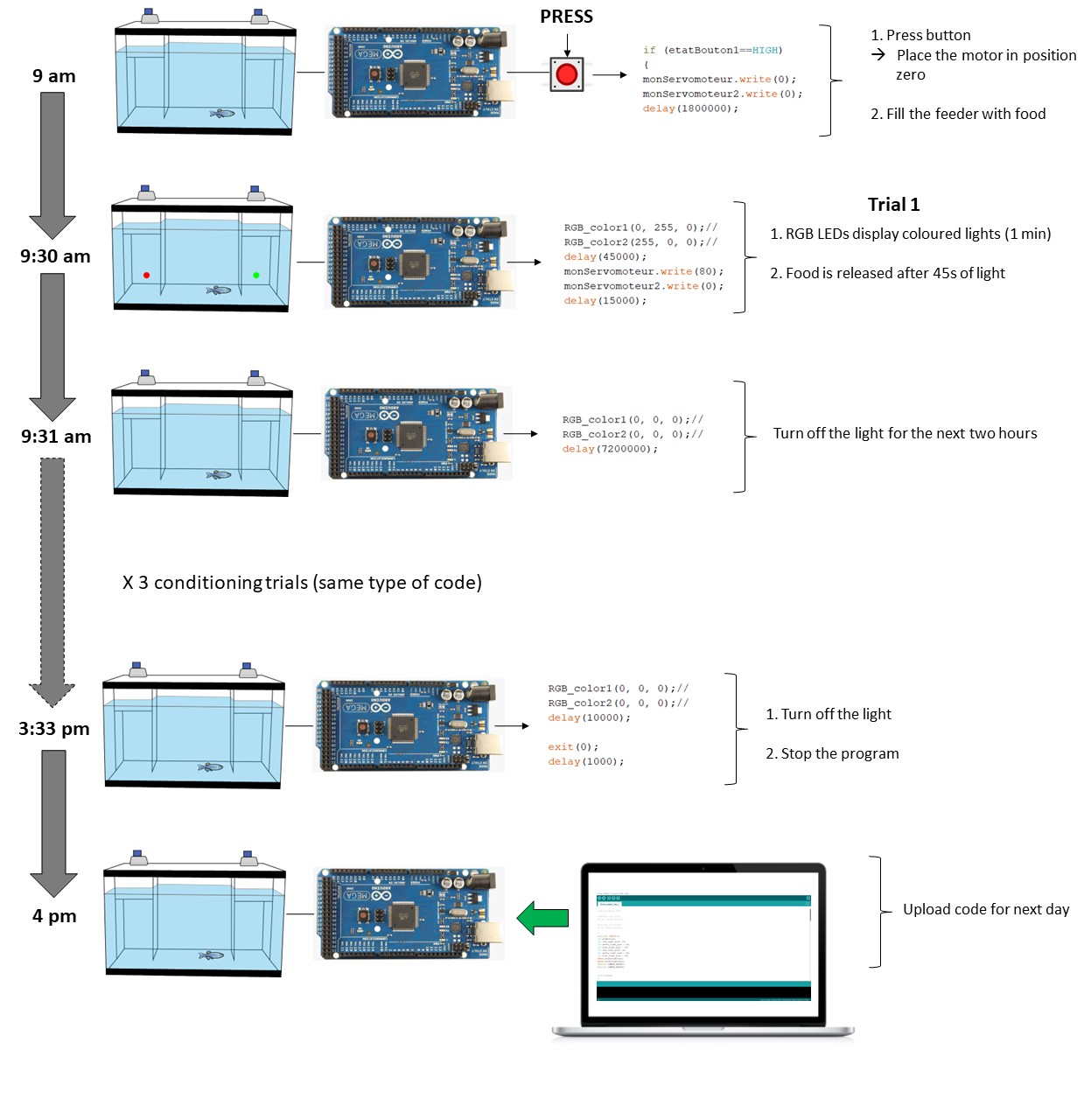
**

**Figure S3. Schedule example of one day of conditioning.** Description of the different step followed by the experimenter and the Arduino card during one conditioning day of discrimination. The experimenter is coming in the room 30 min before to start all this process to prevent a stress induced by its arrival. During this 30 min, the experimenter is checking the health of every experimental fish and feed the fish that are in habituation phase in the habituation tank. At 9 am, the experimenter is pressing the push button to initiate the program in the Arduino card. This is indicated by a positioning of the servomotor in position zero. Then, the experimenter place food in the feeder as described in Fig.S1. From this moment, the experimenter gets out of the experimental room and do not come back before 4 pm. The Arduino then controls the RGB LED and the servo motor following the code that has been uploaded. The experimenter records the first conditioning trial, as well as the test by connecting himself to the cameras from an office next to the experimental room. Once the conditioning is done, the experimenter comes back to the room at 4 pm to check if food was consumed, the health of the fish, feed the fish in the habituation phase, and upload the code in the Arduino card for the next days. Code was changed to ensure that fish does not experience the same sequence of stimuli every day (i.e. for day 1: Green at right during trial 1, at left during trial 2 etc. and for day 2: Green at left during trial 1, at right during trial 2 etc.).

**
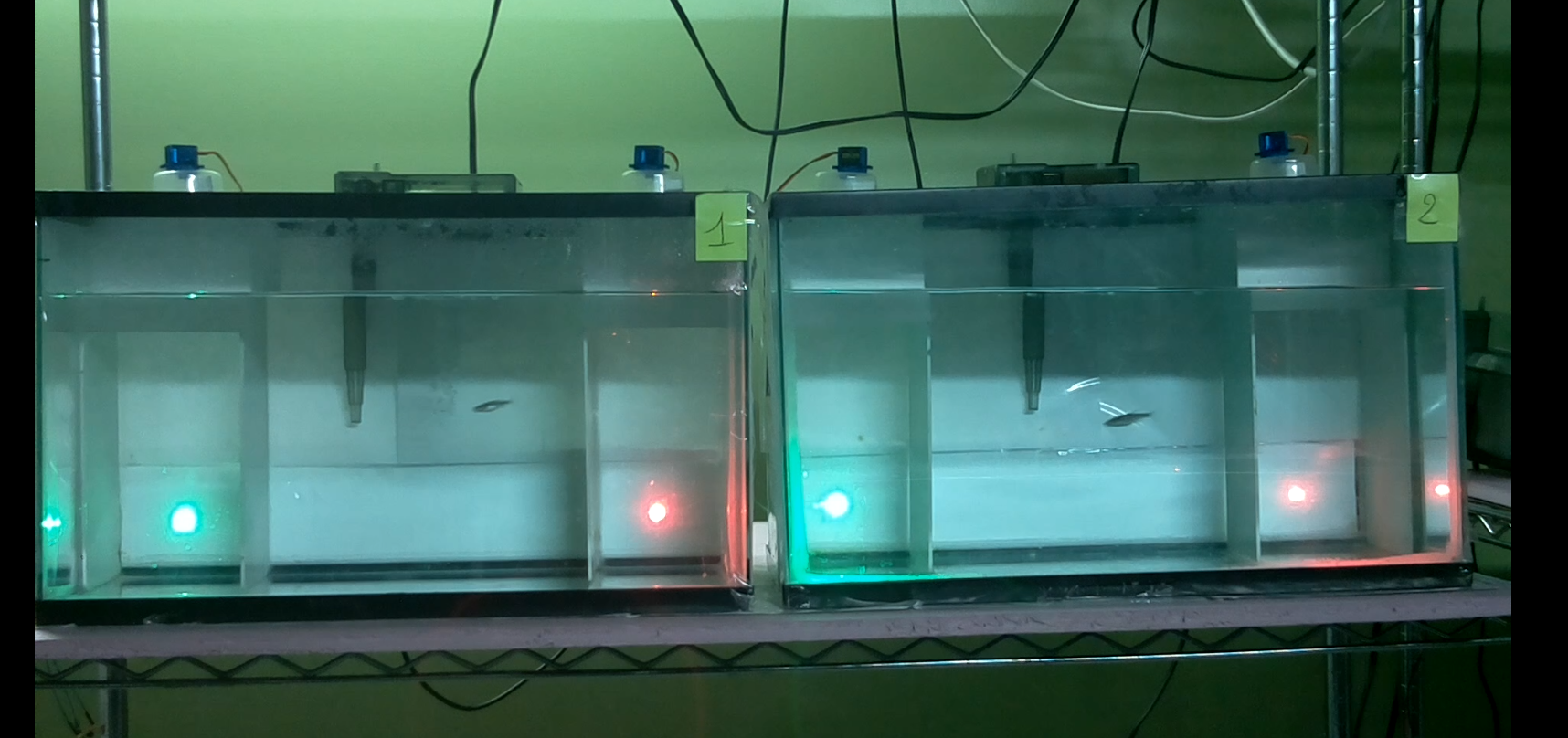
**

**Figure S4. Screenshot of a video recording.** Each camera can record two semi-automatic home-tank. Tanks are covered with white plastic sheets; it is therefore not possible for a fish to see what happens in the aquarium next to it. The camera is placed in front of the tanks and was accessible through WIFI on a computer placed in an office next to the experimental room.


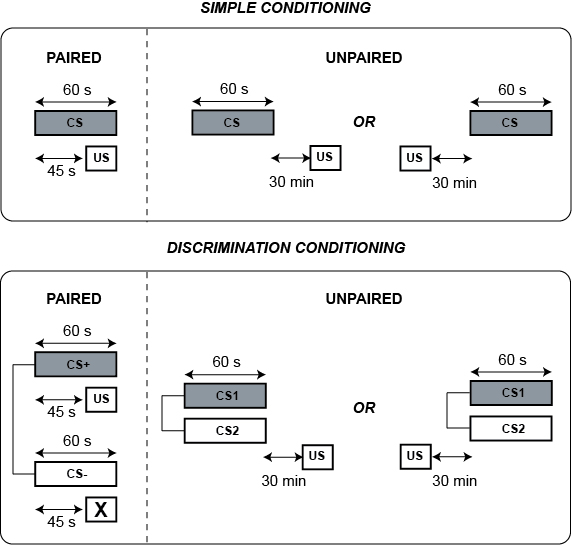


**Figure S5. Conditioning trial timing for simple and discrimination conditioning.** During simple conditioning, fish were separated in two groups: paired and unpaired. In the paired group, for each conditioning trial, the CS (green or red light) was displayed for 60 seconds in one of the two columns. After 45 seconds, the US (food reward) was released in the same column allowing an overlapping of CS-US of 15 seconds. In the unpaired group, the CS was also presented for 60 seconds. However, the US was released 30 min before/after the CS display to unpaired the association CS-US.

During discrimination conditioning, fish were also separated in paired and unpaired groups. In the paired group, CS1 (green light) and CS2 (red light) were displayed for 60 seconds (one per column). After 45 seconds, US (food reward) was released in the same column as one of the CS (chosen in prior to the experiment for having 50/50 rewarded for green and for red light). In this example, CS1 was rewarded and was therefore considered as CS+ and CS2 was not rewarded, and so was considered as CS-. In the unpaired group, both CS1 and CS2 were displayed for 60 seconds (one per column), but reward was randomly release 30 min before or after the coloured-light to unpaired any possible association CS-US.


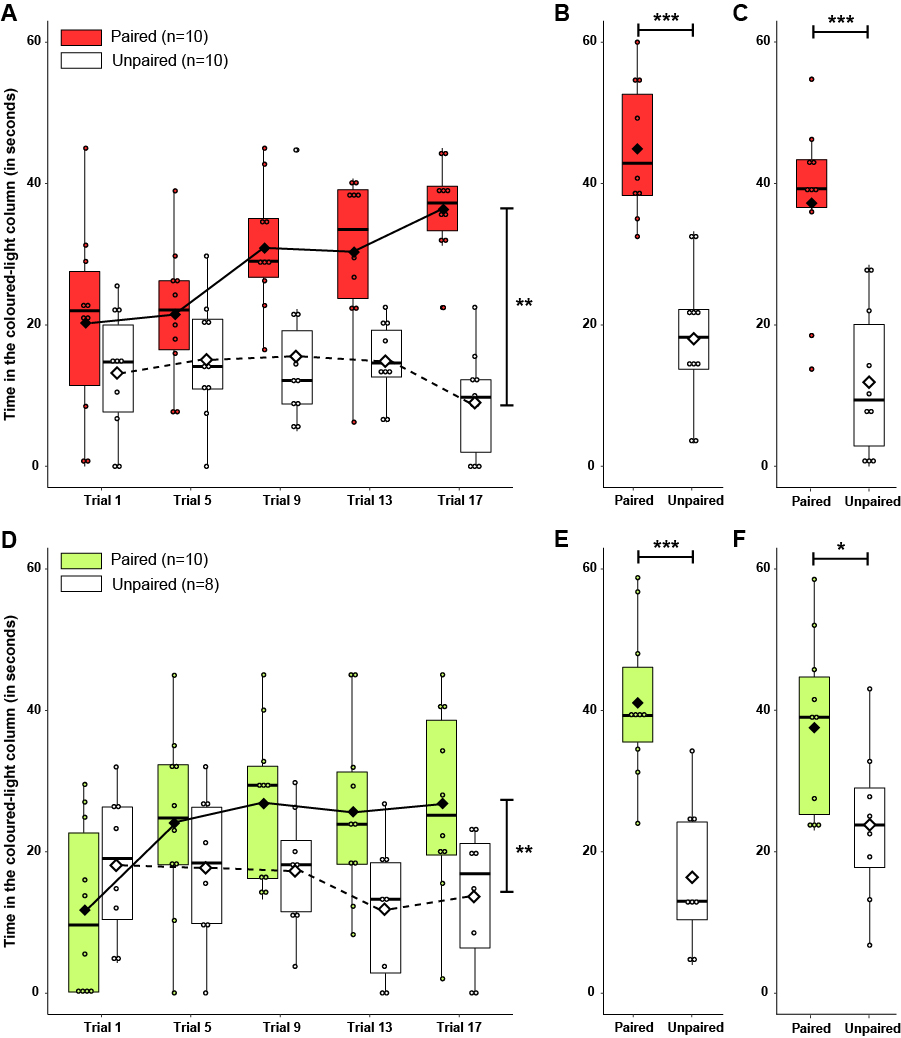


**Figure S6. Simple conditioning. (A)** Time (in seconds, median and quartiles) spent by fish (red-light conditioning) in the column displaying the coloured-light. Data were collected for paired group (n=10, red) and unpaired group (n=10, white) during the first 45 seconds of coloured-light display of the first conditioning trial of each conditioning day. **(B)** Time (in seconds, median and quartiles) spent by fish (red light conditioning) from paired group (n=10, red) and unpaired group (n=10, white) in the coloured-light column during the 60 seconds of the memory test. **(C)** Time (in seconds, median and quartiles) spent by fish (red light conditioning) from paired group (n=10, red) and unpaired group (n=10, white) in the coloured-light column during the 60 seconds of the generalization test. **(D)** Time (in seconds, median and quartiles) spent by fish (green light conditioning) in the column displaying the coloured-light. Data were collected for paired group (n=10, red) and unpaired group (n=8, white) in the coloured-light column during the first 45 seconds of coloured-light display of the first conditioning trial of each conditioning day. **(B)** Time (in seconds, median and quartiles) spent by fish (green light conditioning) from paired group (n=10, red) and unpaired group (n=8, white) in the coloured-light column during the 60 seconds of the memory test. **(C)** Time (in seconds, median and quartiles) spent by fish (green light conditioning) from paired group (n=10, red) and unpaired group (n=8, white) in the coloured-light column during the 60 seconds of the generalization test. **(A,B,C,D,E,F)** Small dots represent individual data, and diamond shape connecting the curve represent the mean time for each group. *p < 0.05; **p < 0.001, ***p < 0.0001; NS, non-significant.


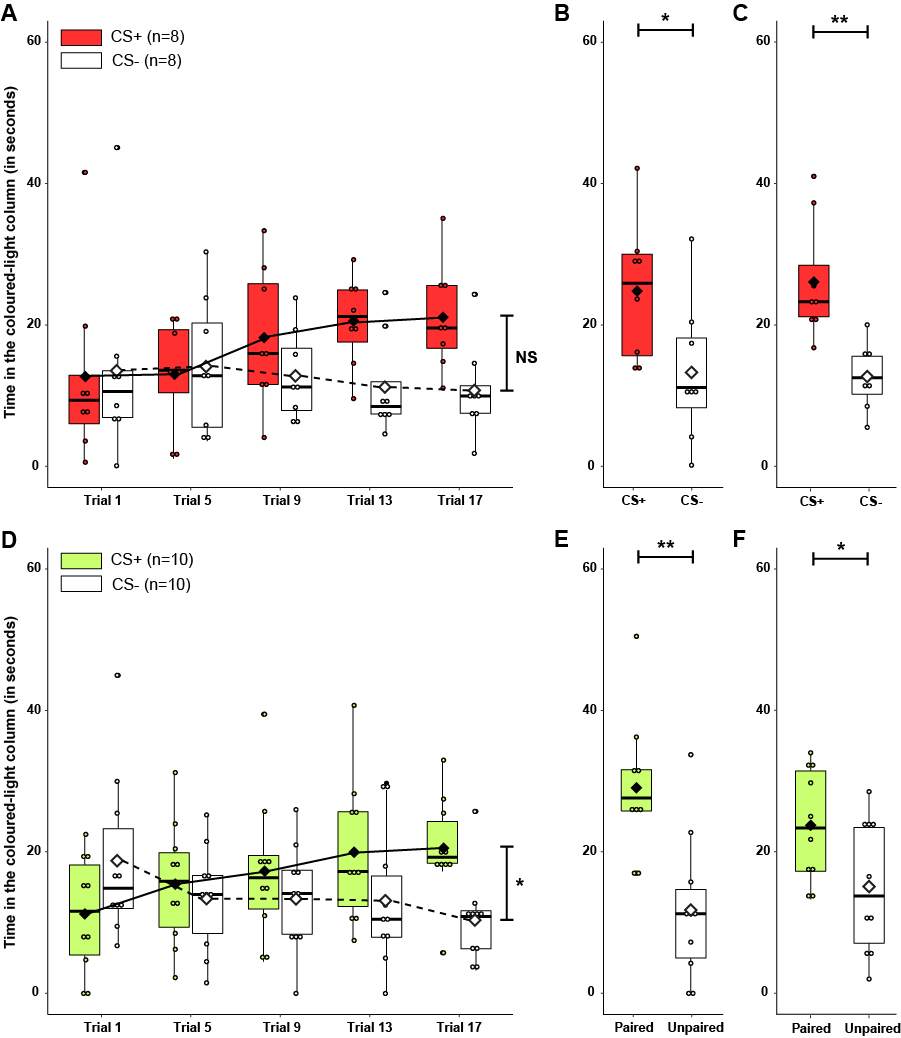


**Figure S7. Discrimination conditioning. (A)** Time (in seconds, median and quartiles) spent by fish rewarded for red coloured-light from paired group (n=8) in the CS+ (red) and CS- (white) columns during the 45 seconds preceding the food reward release during the first conditioning trial of each conditioning day. **(B)** Time (in seconds, median and quartiles) spent by fish rewarded for red coloured-light from paired group (n=8) in the CS+ (red) and CS- (white) columns during the 60 seconds of the first memory test. **(C)** Time (in seconds, median and quartiles) spent by fish rewarded for red coloured-light from paired group (n=8) in the CS+ (red) and CS- (white) columns during the 60 seconds of the second memory test. **(D)** Time (in seconds, median and quartiles) spent by fish rewarded for green coloured-light from paired group (n=10) in the CS+ (green) and CS- (white) columns during the 45 seconds preceding the food reward release during the first conditioning trial of each conditioning day. **(B)** Time (in seconds, median and quartiles) spent by fish rewarded for green coloured-light from paired group (n=10) in the CS+ (green) and CS- (white) columns during the 60 seconds of the first memory test. **(C)** Time (in seconds, median and quartiles) spent by fish rewarded for green coloured-light from paired group (n=10) in the CS+ (green) and CS- (white) columns during the 60 seconds of the second memory test. **(A,B,C,D,E,F)** Small dots represent individual data, and diamond shape connecting the curve represent the mean time for each group. *p < 0.05; **p < 0.001, ***p < 0.0001; NS, non-significant
